# Post-Translational Modifications Modulate Proteinopathies of TDP-43, FUS and hnRNP-A/B in Amyotrophic Lateral Sclerosis

**DOI:** 10.3389/fmolb.2021.693325

**Published:** 2021-07-05

**Authors:** Stefania Farina, Francesca Esposito, Martina Battistoni, Giuseppe Biamonti, Sofia Francia

**Affiliations:** ^1^ Istituto di Genetica Molecolare “Luigi Luca Cavalli-Sforza” - Consiglio Nazionale delle Ricerce (CNR), Pavia, Italy; ^2^ University School for Advanced Studies IUSS, Pavia, Italy; ^3^ Università Degli Studi di Pavia, Pavia, Italy

**Keywords:** post-translational modifications, RNA binding proteins, low-complexity domain, protein aggregations, amyotrophic lateral sclerosis

## Abstract

It has been shown that protein low-sequence complexity domains (LCDs) induce liquid-liquid phase separation (LLPS), which is responsible for the formation of membrane-less organelles including P-granules, stress granules and Cajal bodies. Proteins harbouring LCDs are widely represented among RNA binding proteins often mutated in ALS. Indeed, LCDs predispose proteins to a prion-like behaviour due to their tendency to form amyloid-like structures typical of proteinopathies. Protein post-translational modifications (PTMs) can influence phase transition through two main events: i) destabilizing or augmenting multivalent interactions between phase-separating macromolecules; ii) recruiting or excluding other proteins and/or nucleic acids into/from the condensate. In this manuscript we summarize the existing evidence describing how PTM can modulate LLPS thus favouring or counteracting proteinopathies at the base of neurodegeneration in ALS.

## Introduction

In healthy organisms, proteins are properly folded into secondary and tertiary structures suited to their biological functions. However, mutations, cellular stress and aging can perturb protein structure leading to the formation of insoluble protein aggregates. Although it is now well established that protein aggregation is a common hallmark of several neurodegenerative diseases including Amyotrophic Lateral Sclerosis (ALS), Frontotemporal Dementia (FTD) and Alzheimer’s disease (AD), the pathological mechanisms that drive their formation are still uncertain (Aguzzi and O'Connor, 2010). Indeed, neurodegenerative disorders are widely defined as proteinopathies, which refers to the fact that these diseases are characterized by the accumulation of protein aggregates in the brain and/or spinal cord of patients (Forman et al., 2004; Ross and Poirier, 2004; Chiti and Dobson, 2006).

Protein aggregation is believed to originate from the alteration of the physiological propensity of some proteins to undergo liquid-liquid phase separation (LLPS), i.e., a transient and normally reversible phase transition that separates two liquid compartments with different viscosity and composition (Posey et al., 2018). LLPS generates cellular condensates, organelles with a biological function but not delimited by a lipid membrane. In the last decade a number of physiological cellular condensates have been characterized, some of which have been purified and their components and modifications identified by mass spectrometry. Examples are nucleoli, speckles and paraspeckles, nuclear stress bodies, P-granules, stress granules (SGs) and Cajal bodies (Toretsky and Wright, 2014).

A common feature of proteins with the propensity to undergo LLPS is the presence of low complexity domains (LCDs), which exhibit a high level of conformational heterogeneity. The structural plasticity of LCDs makes them ideal for responding to chemical and physical changes, thus providing the potential of rapid tuning of localized molecular functions (van der Lee et al., 2014). Human proteins holding LCDs have features in common with prion proteins, such as the ability to induce mis-folding in interacting peptides, thus propagating proteinopathies within the cells (Alberti et al., 2009) and in the surrounding tissues, eventually affecting big areas of the nervous system (Jucker and Walker, 2013). Typically, LCDs are enriched in charged amino acids, including serine (Ser), glutamine (Gln), glutamic acid (Glu), lysine (Lys) and arginine (Arg) (Romero et al., 2001), which form Arg-Gly-Gly/Arg-Gly (RGG/RG) motifs in a large number of proteins, mostly RNA binding proteins (RBPs) (Thandapani et al., 2013). Moreover, the sequences that drive the formation of condensates often contain regularly interspersed aromatic residues, specifically tyrosine (Tyr) and phenylalanine (Phe) that mediate π-interactions. Depending on the amino acid composition of the LCDs, charge-charge, charge-π hydrogen bonding and π-π stacking interactions can be established between two residues. In the first case, the interaction is between two residues with opposite charges. In the second situation, one positive charge interacts with a negative charge distributed above an aromatic group. In the third case, two aromatic groups are positioned above each other in a stacked conformation (Brangwynne et al., 2015).

RBPs represent a large group of proteins undergoing LLPS, and phase transition is modulated by their secondary structure and by the concentration of RNA (Langdon and Gladfelter, 2018; Roden and Gladfelter, 2021). Indeed, several cellular condensates include an RNA moiety with a structural role. The RNA (which can also coalesce into droplets) provides a multivalent binding site for the interaction with different RBPs, thus promoting further contacts between their LCDs. Different regulatory circuits take advantage of the inherent property of LCDs to induce separate cellular sub-compartments and to tightly modulate phase transition upon specific stimuli and activation of signaling cascades. LCDs, in fact, are preferred targets of post-translational modifications (PTMs) (Dosztányi et al., 2006; Xie et al., 2007; Wright and Dyson, 2009) that can promote or inhibit protein-protein and protein-RNA interactions, thus modulating possible changes in protein compartmentalization and sequestrations.

Here we review different PTMs that finely regulate the biophysical properties of RiboNucleoProtein A and B type (hnRNP-A/B type meaning hnRNP-A1 and hnRNP-A2), Trans-activating response (TAR) element DNA-binding protein of 43 kDa (TDP-43) and Fused in Sarcoma (FUS) in the attempt to in an attempt to shed light on common paradigms that can modulate the pathological phase transition in the context of neurodegeneration. Similarly to other protein functions, PTMs can alter phase transition and protein aggregation in different ways by both stimulating and counteracting it, depending on their charge, the amino acid residue that is modified and its position in the target proteins (Owen and Shewmaker, 2019). A lot still needs to be understood regarding how PTMs can cause or prevent pathological aggregation and proteinopathies. This review aims at summarizing recent studies that describe the impact that specific PTMs have on biophysical properties of three RBPs relevant to ALS: hnRNP-A1 and hnRNP-A2 TDP-43 and FUS.

## hnRNP-A1, TDP-43 and Fus Protein Structure and Function

**FIGURE 1:**
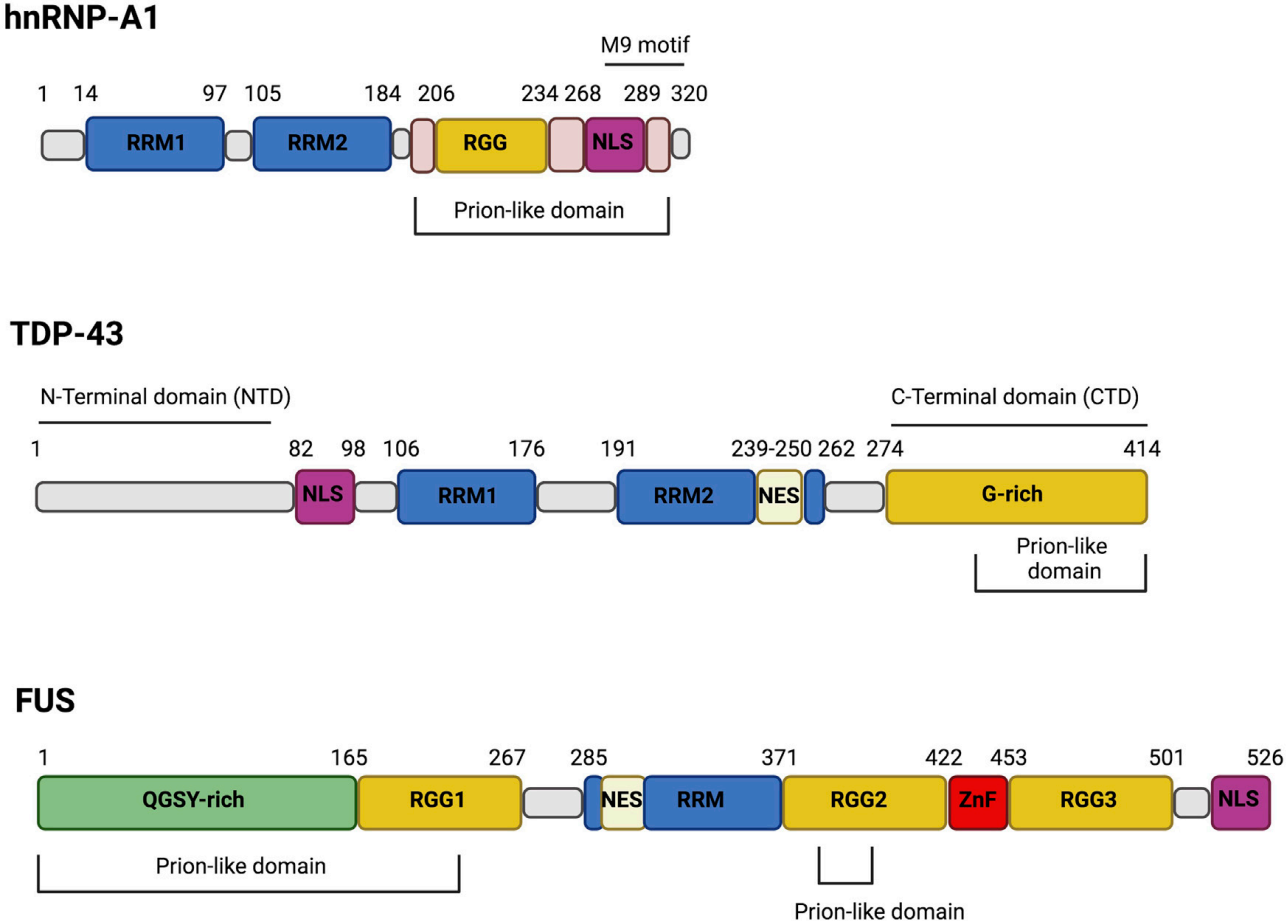
The domain structure of hnRNP-A1, TDP-43 and FUS. The RNA-binding proteins hnRNP-A1, TDP-43 and FUS share structure similarities. Particularly, they harbor Prion-like domain, RNA-recognition motif (RRM) and nuclear localization signal (NLS). hnRNP-A1 and FUS are both characterized by the presence of Arg-Gly-Gly-rich (RGG) domains while TDP-43 has a Gly-rich (G-rich) domain and hnRNP-A1 display a M9 motif. Moreover, both FUS and TDP-43 present nuclear export signal (NES) but only FUS has a zinc-finger (ZnF) domain and a Gln-Gly-Ser-Tyr-rich (QGSY-rich) domain.

The hnRNP-A1 protein is the founding member of the A/B group of hnRNPs. These proteins share a common organization consisting of two RNA recognition motifs (RRMs) at the N-terminus followed by a C-terminal LCD that contains RGG/RG repeats (Figure 1). The second half of the LCD harbors the nuclear localization signal (NLS) sequence (Figure 1) that displays a high affinity for Karyopherin-β2 and controls the distribution of these nucleo-cytoplasmic shuttling proteins. Missense mutations in the LCD are causatively linked to ALS and multisystem proteinopathy (MSP) (Bosco et al., 2010; Hackman et al., 2013; Kim et al., 2013). The LCD is sufficient to drive LLPS of hnRNP-A1. However, the two RRMs contribute to phase separation by binding RNA molecules, whose polymeric structure increases the local protein crowding and lowers the protein concentration required to form LLPS (Molliex et al., 2015).

Biophysical analyses indicate that Arg and aromatic residues (Phe and Tyr), evenly distributed throughout the LCD, play a major role in LLPS by producing a repeated motif that enables multivalent interactions (Molliex et al., 2015). The hnRNP-A1 protein exists in three assembly states: liquid-like droplets, reversible fibrils, and irreversible fibrils. While the first two forms are physiological, the latter assembly is ALS-related and corresponds to a highly ordered stacking of proteins that is very difficult to disassemble. Three segments, each containing Asn-Asp-Asn and (Gly)Phe/Tyr(Gly) motifs separated by Arg/Gly rich stretches, have been mapped within the LCD. Each segment is able to assist the formation of reversible fibrils and hydrogels. Asp residues have a key role in the reversibility of amyloid formation, which explains why the disease-linked mutations of these residues enhance irreversible amyloid aggregation and pathogenesis of ALS (Gui et al., 2019).

TDP-43 and FUS are two RBPs that contain LCDs and undergo phase separation (Conicella et al., 2016; Molliex et al., 2015; Patel et al., 2015; Schmidt and Rohatgi, 2016). TDP-43 has been found in cytosolic aggregates in many neurodegenerative diseases, including ALS, FTD and limbic-predominant age-related TDP-43 encephalopathy (LATE) (de Boer et al., 2020). Similarly to hnRNP-A1, TDP-43 contains two RRMs followed by a C-terminal domain (CTD) that is mostly disordered and enriched in Arg and Gly residues with a regular spacing of hydrophobic residues (Val, Leu, Ile, Met, Phe, Tyr, Trp). However, unlike hnRNP-A1, TDP-43 contains another folded N-terminal domain (NTD) as well (Figure 1). The CTD has a central role in determining the functional properties of the protein, since it controls most of its interactions and sub-cellular distribution by regulating the nucleo-cytoplasmic shuttling. Moreover, the CTD has been widely described as the main contributor to LLPS (Conicella et al., 2016; Lim et al., 2016; Schmidt and Rohatgi, 2016). It is, therefore, not surprising that most of ALS-associated mutations in TDP-43 map to this domain. The CTD seems particularly involved in TDP-43 aggregation, especially the glycine-rich region (G-Rich Figure 1) that contains three different amyloidogenic cores: residues 286–331, 318–343 and 342–366. The amyloidogenic core 318–343 includes a hydrophobic patch (HP) and a Gln/Asn (QN)-rich motif (Jiang et al., 2013). Deletion of the HP or the QN region reduces the ability of TDP-43 to form aggregates (Jiang et al., 2013). The 318–343 peptide is composed of two α-helices connected by a turn of 4 amino acids (Jiang et al., 2013), forming a helix-turn-helix structure. This structure can generate an antiparallel hairpin-like β-sheet, which can interact with other TDP-43 molecules leading to aggregation. According to Saini and Chauhan, the initial deca-peptide (311–320) of this helix-turn-helix is necessary for TDP-43 aggregation and loss of this region abrogates the formation of inclusions. Furthermore, another deca-peptide (246–255) within RRM2 has been identified as an important region for TDP-43 aggregation, even though its deletion does not completely abolish the formation of TDP-43 filaments (Saini and Chauhan, 2011). Both Ala^324^Glu and Met^337^Glu mutants, located in the hydrophobic region, introduce negative charges reducing the ability of TDP-43 to form aggregates (Jiang et al., 2016). The Gln^343^Arg mutation present in familial ALS (fALS) cases also reduces TDP-43 aggregation by generating a single α-helix that is not stackable into a β-sheet. On the contrary, the Gly^335^Asp mutation, that has high frequency in Italian ALS patient, causes an increase of amyloidogenic aggregation, due to an extension of the loop in the helix-loop-helix (Jiang et al., 2016). Finally, the mutation Ala^315^Thr in the CTD has been proposed to increase the ability of TDP-43 to form β-sheet (Guo et al., 2011). The pronounced sensitivity of TDP-43 LLPS to single amino acid substitutions with different properties is consistent with the fact that single PTMs on specific residues can strongly impact on phase transition.

FUS, also known as hnRNP P2, is a member of the FET family together with the EWS protein, the TATA-binding protein (TBP)-associated factor (TAFII68/TAF15) and the *Drosophila* cabeza/SARF protein. FUS is a nucleo-cytoplasmic shuttling RBP formed by an N-terminal LCD rich in Gln-Gly-Ser-Tyr (QGSY), an RRM, three RGG repeats, a zinc-finger (ZnF) motif, and C-terminal NLS (Guerrero et al., 2016). Both TDP-43 and FUS are mainly nuclear; nevertheless their insoluble aggregates are cytosolic. The nuclear localization of FUS relies on a non-canonical NLS in the C-terminus of the protein (residues 514–526) that mediates the interaction with the nuclear import receptor transportin (TRN) (Chook and Süel, 2011). FUS mutations in familial ALS/FTD patients are mostly located in the NLS, leading to its cytoplasmic mislocalization and inclusion formation. Cytoplasmic localization, however, although required is not sufficient to promote aggregation. The ability of FUS to undergo LLPS relies on the N-terminal LCDs and PTMs that occurs in the QGSY-rich patch (Figure 1) also affects it (Burke et al., 2015; Murakami et al., 2015; Patel et al., 2015; Shorter, 2017). As for other prion-like proteins, the LCD of FUS appears predominantly disordered in reversible condensates (Burke et al., 2015) whereas it is well organized in packed β-sheets when forming irreversible aggregates (Hughes et al., 2018). Specific mutations in the LCD or the NLS of FUS increase the total protein levels, a condition that may enhance the conversion of FUS condensates from liquid to a solid state (Guerrero et al., 2016). The altered subcellular distribution also changes the interactions of FUS with specific RNA subsets, with the cytoplasmic mutants binding more frequently to the 3^′^ UTRs of target mRNAs instead of nuclear intronic sequences (Hoell et al., 2011).

**FIGURE 2:**
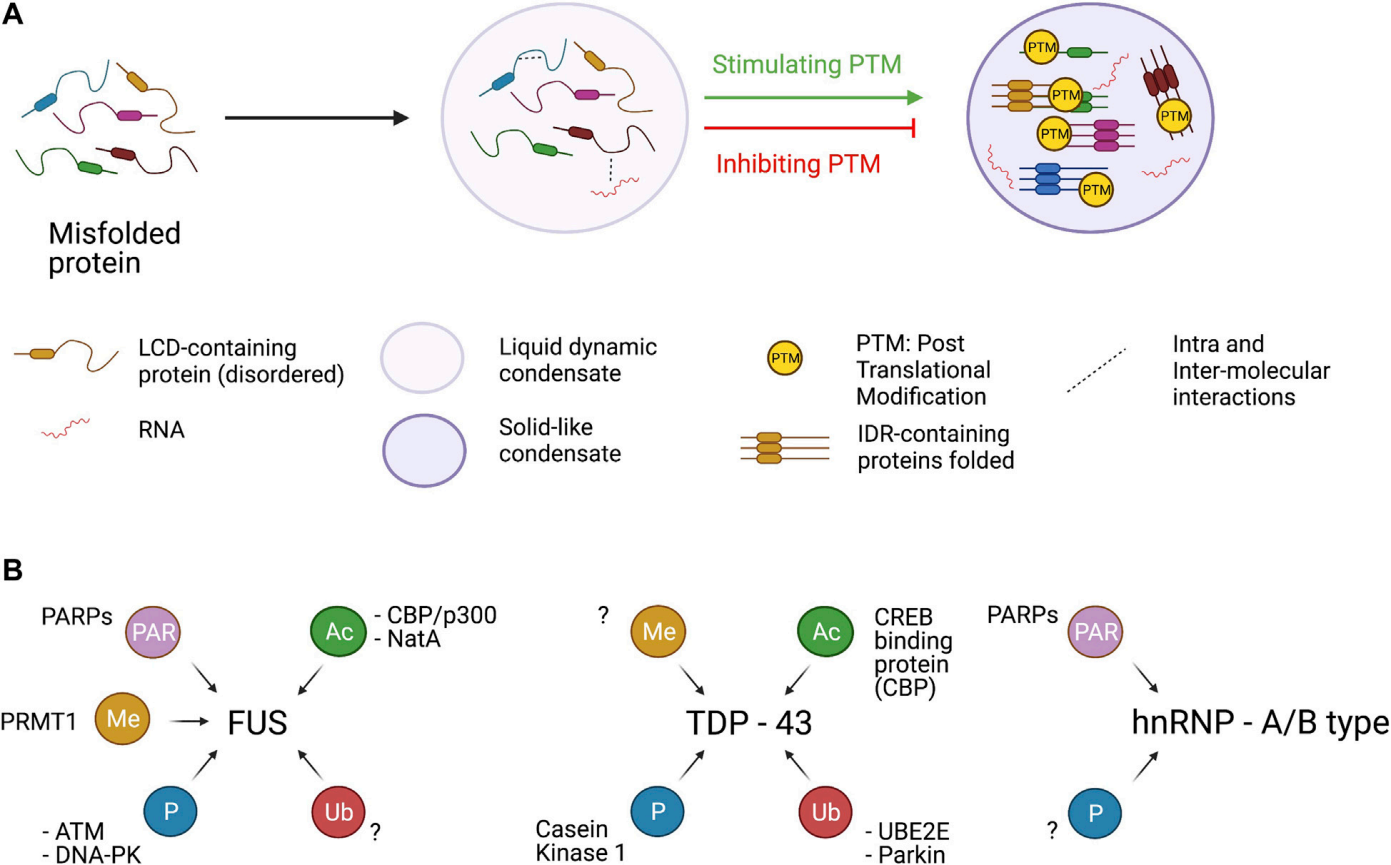
Assembly of condensates and PTMs. **Panel A**. LCD-containing proteins (including TDP-43, FUS and hnRNPA1) under certain conditions assume a misfolded state in which several intra and inter-molecular interaction can be established. Different PTMs tend to stimulate or dampen the formation of insoluble condensate in which LCD-containing proteins are sequestered in a toxic β-sheet structure. However, PTM has protein- and residue-specific impact on protein aggregate formation. **Panel B**. hnRNPA1, FUS and TDP-43 can be target of several PTMs catalyzed by specific enzymes. hnRNP-A1 and FUS can be PARylated by PARPs and PARP1, respectively. FUS protein can be methylated by PRMT1 acetylated by CBP/p300 and NatA. Moreover, the Prion like domain of FUS is phosphorylated at multiple sites by the two kinases ATM and DNA-PK. TDP-43 is actively acetylated by CBP and phosphorylated by Casein Kinase. Finally, UBE2E and Parkin catalyze the ubiquitination of TDP-43.

All these observations support the idea that FUS condensates are in equilibrium between liquid or gel-like states which are both physiological and can alternate each other or even co-exist. When an event perturbs this equilibrium (e.g., familial ALS mutations or reduction in R-methylation state) FUS condensate can shift to a more pathological solid-like state (Figure 2A).

Recently, TDP-43 and FUS have been shown to contribute to DNA repair (Rulten et al., 2014; Naumann et al., 2018; Wang H. et al., 2018; Mitra et al., 2019; Singatulina et al., 2019; Levone et al., 2020). FUS is recruited to DNA damage sites in a PARP-1 dependent manner (Rulten et al., 2014) via the interaction with HDAC1 (Wang et al., 2013) and is phosphorylated by ATM and DNA-PK (Monahan et al., 2017; Rhoads et al., 2018a). The recruitment of FUS at sites of DNA lesions correlates with PARP-1 dependent FUS PARylation required for DNA repair (Singatulina et al., 2019). Moreover, *in vitro* studies have shown that the addition of purified PAR strongly stimulates the formation of FUS-containing droplets essential for the proper activity of FUS in DNA repair mechanism (Patel et al., 2015). TDP-43 can interact with the sensor protein KU70 at sites of DNA damage, suggesting that it plays a role in the non-homologous end joining (NHEJ) mechanism (Freibaum et al., 2010). Furthermore, TDP-43 can interact with other factors of the DNA damage response (DDR), such as DNA-PK and 53BP1 that are being recruited at DNA damage sites during NHEJ (Mitra et al., 2019).

## Post-Translational Modifications of TDP-43, Fus and hnRNP-A1 Controlling Liquid-Liquid Phase Separation and Protein Aggregation

The role of PTMs in regulating the propensity of LCD-containing proteins to undergo LLPS is an emerging area of study due to its possible therapeutic impact. To date, several PTMs have been described for hnRNP-A1, TDP-43 and FUS but we will discuss mostly the ones that modulate LLPS. These are: protein methylation, phosphorylation, acetylation, ubiquitination and PARylation (Rhoads et al., 2018b; Buratti, 2018).

### Arginine Methylation

Several proteins involved in DNA and RNA metabolism, including histones and a large number of RBPs, undergo Arg-methylation, which may occur in different flavors (mono, symmetric and asymmetric di-methylation) (Blanc and Richard, 2017). In mammalian cells, Arg (R)-methylation is catalyzed by at least nine Protein-Arginine-Methyl-Transferases (PRMTs), from 1 to 9 (Bedford and Clarke, 2009; Blanc and Richard, 2017). Of these, at least three (PRMT1, 6 and 8) catalyze asymmetrically di-methylation on arginine (ADMA). PRMT1 catalyses the addition of one or two methyl groups to the R residues from the S-adenosylmethionine donor (Yang and Bedford, 2013). Methylation plays a major role in controlling the biophysical properties of RGG/RG motifs and modulates both protein-protein and RNA-protein interactions. Notably, arginine methylation is very relevant for FET proteins (Figure 2B) (Lorton and Shechter, 2019) while not many studies have characterized the impact of arginine methylation in TDP-43 so far, even though some arginine methylated residues had been identified and reported in databases. Indeed, in the phosphosite.org website it is reported that mass spectrometry analyses identified three TDP-43 methylated residues: Arg 42, 275 and 293. The last two modifications are located at the C-terminal domain of TDP-43, thus could in principle influence LLPS, however the functions of these modifications and their regulation have not been investigated yet (Figure 2B). Methylation does not alter the net charge of the protein, but changing its distribution can regulate the capacity of Arg to enter cation-π interactions with aromatic residues (Lorton and Shechter, 2019) and hence the ability of FUS to rapidly and reversibly form liquid droplets and hydrogels (Figure 2A). In fact, LLPS transition of FUS involves hydrogen bonding between an antiparallel β-sheet in the LCD (residues 39–95) and Arg residues in the three RGG-rich regions (Han et al., 2012; Murakami et al., 2015; Patel et al., 2015; Murray et al., 2017). Under normal conditions these Arg residues are heavily mono- or di-methylated (Rappsilber et al., 2003). In contrast, in FTLD they are hypomethylated and FUS is found in neuronal nuclear and cytoplasmic aggregates that frequently contain other members of the FET family. Several lines of evidence indicate that methylation of specific Arg residues (position 216, 259, 407, 472, 473 and 476) has an inhibitory effect on condensate formation (Qamar et al., 2018). Indeed, inhibition of arginine methyltransferase activities with Adenosine dialdehyde (AdOx) produces a significant reduction in asymmetrical di-methylation of FUS at most of these Arg (216, 259, 407, 473, and 476) and promotes LLPS. In contrast, Arg 394 and 481 remain predominantly di-methylated, indicating that a higher methylation turnover occurs only in the case of Arg residues involved in LLPS, allowing dynamically tuning phase separation. Altogether these data suggest that the number of methylated Arg residues can modulate the type of phase separation (liquid-liquid vs liquid-solid), which is driven by multivalent cation-π interactions. FUS is normally soluble and dimethylated in healthy brains while reduced levels of FUS methylation have been detected in insoluble protein inclusions in brains of FTD patients (Suárez-Calvet et al., 2016).

Paradoxically R-methylation can also promote the formation of aggregates (Table 1). Indeed, recent studies demonstrate that R-methylation in the non-canonical NLS domain of FUS influences the subcellular distribution of the protein. This is due to the fact that methylation of Arg residues in the second RGG-rich region of FUS (Figure 1) abrogates the interaction of TRN with the third RGG-rich region thus reducing FUS nuclear import and increasing its cytoplasmic concentration, thus favoring LLPS (Dormann et al., 2012). Indeed, cell treatment with methylation inhibitors or PRMT1 knock down can restore the nuclear localization of the ALS-linked FUS mutant protein Pro^525^Lys (Dormann et al., 2012). Interestingly, immunohistochemical analysis of FUS-Pro^525^Lys ALS patients revealed the presence of inclusions with methylated FUS that are undetectable in FTD patients (Dormann et al., 2012). Thus, detection and quantification of methylated forms of FUS can be a valuable biomarker of ALS and not of FTD.

**TABLE 1:** PTMs and their effects on RBP’s aggregation.

**PTM**	**RBP**	**Residue**	**Effects on Aggregation**	**References**
**R-methylation**	FUS	Arg 216, Arg 259	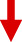	Qamar et al. (2018)
		Arg 407, Arg 472		
		Arg 473, Arg 476		
**Phosphorylation**	FUS	Ser 26/Ser 30	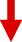	Deng et al. (2014), Monahan et al. (2017)
		Ser 30/Ser42		Rhoads et al. (2018a)
		Thr 109/Ser 115		
		Ser 115/Ser 117		
	hnRNP - A2	Tyr (n.d.)	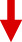	Ryan et al. (2021)
	TDP - 43	Ser 48	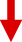	Hornbeck et al. (2012), Hornbeck et al. (2015)
				Rigbolt et al. (2011), Wang A. et al. (2018)
	TDP - 43	Ser 403/404	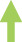	Neumann et al. (2020)
		Ser 409/410		
**Acetylation**	FUS	Lys 510	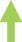	Arenas et al. (2020)
	FUS	Lys 315/316	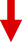	Arenas et al. (2020)
	FUS	Ala 2	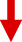	Bock et al. (2021)
	TDP - 43	Lys 145–149	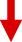	Cohen et al. (2015)
**Ubiquitination**	TDP - 43	Lys 48, Lys 63	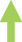	Hebron et al. (2013)
	TDP - 43	Lys 263	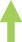	Hans et al. (2014)
**PARylation**	hnRNP - A1	Lys 298	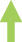	Duan et al. (2019)
	FUS	n.d	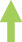	Patel et al. (2015)

PTMs are able to both suppress (red arrow down) and enhance (green arrow up) protein aggregation of FUS, TDP43 and hnRNP-A1/A2. R-methylation mainly suppresses FUS aggregation while ubiquitination and PARylation stimulates aggregation of TDP-43, hnRNP-A1 and FUS. Other PTMs such as phosphorlation and acetylation, have been shown to suppress or enhance aggregation propensity depending on the specific residue and protein modified.

Interestingly, methylation events have been shown to affect the nucleo-cytoplasmic trafficking of other RBPs such as hnRNP-A2 (Nichols et al., 2000) and binding of hnRNP-A1 to single-stranded nucleic acid is significantly reduced after arginine methylation (Rajpurohit et al., 1994).

Very little is still known about the mechanisms by which R-methylation can be erased. Recent data suggest the involvement of R-demethylating enzymes such as KDM3A, KDM4E, KDM5C (Walport et al., 2016) and JMJD6 (Chang et al., 2007), all belonging to the large family of 2-oxoglutarate-dependent dioxygenases. Numerous studies indicate that some RGG motifs are protected from methylation, while other motifs are preferentially recognized by the methylating enzymes. The molecular basis of this difference, however, is still a matter of speculation.

Interestingly, Arg residues in the RGG motifs of FET proteins and hnRNP-A1 can also undergo citrullination, catalyzed by peptidyl arginine deiminase 4 (PAD4), which significantly inhibits protein aggregation and the recruitment of FUS in arsenite-induced stress granules. In agreement with this, a lower PAD4 expression is associated with a higher risk of developing ALS (Tanikawa et al., 2018).

Contrary to methylation, phosphorylation and acetylation change the protein charge with consequent impact on proteins conformation (Hofweber and Dormann, 2019) and pattern of interaction, aspects that we will discuss in the following paragraphs.

### Phosphorylation

A complex interplay between protein phosphorylation and methylation has been recently found to control the dynamics of some RBPs including hnRNP-A1 and TDP-43. For instance, cisplatin treatment (CDDP) induces phosphorylation of protein methyl-transferase PRMT1 by DNA-PK, which redirects PRMT1 activity toward chromatin-associated proteins at the cost of RBP methylation (Musiani et al., 2020). Interestingly, 82% of the down-regulated Arg-methyl sites following PRMT1 phosphorylation by DNA-PK are inside the RGG-containing LCDs of proteins undergoing LLPS. As described above Arg-methylation by PRMT1 on these proteins weakens cation–π interactions between Arg and aromatic (Phe and Tyr) residues, thus reducing LLPS. The effect of DNA-PK on this phenomenon is double. In fact, in addition to phosphorylating and redirecting PRTM1 toward chromatin, DNA-PK phosphorylates the RBPs that are a target of R-methylation in a way that inhibits their interaction with PRTM1. The net effect is that these RBPs accumulate in SG condensates (Giambruno and Bonaldi, 2020). In the case of hnRNP-A2, Tyr-phosphorylation alters the propensity of the protein to undergo LLPS *in vitro*, prevents partitioning of granule components and hinders aggregation of mutants associated with neurodegenerative disorders. Moreover, different phosphorylation events in the same domain may elicit different effects offering the possibility of tuning protein assemblies (Ryan et al., 2021). *C. elegans* experiments have identified FYN kinase as a candidate for hnRNP-A2 phosphorylation (Ryan et al., 2021). Indeed, Tyr phosphomimetic mutations, i.e., substitutions with aspartic or glutamic acid that mimic the phosphate negative charge, prevent partitioning in droplets of hnRNP-F and ch-TOG, two molecular partners of hnRNP-A2, while Ser phosphomimetic ones do not (Ryan et al., 2021).

Similarly to R-methylation, phosphorylation can either enhance or suppress LLPS of RBPs *in vitro* (Table 1), as clearly demonstrated for both FUS and TDP-43. In response to DNA damage the two apical DDR kinases DNA-PK and ATM catalyze the phosphorylation of different sites (Ser-26/Ser-30, Ser-30/Ser-42, Thr-109/Ser-115, and Ser-115/Ser-117 within the ATM and DNA-PK consensus Ser/Thr-Gln) localized in the LCD of FUS, a modification that has been shown to prevent liquid to-solid-state transition and the formation of fibril-like structures (Deng et al., 2014; Monahan et al., 2017; Rhoads et al., 2018a). Although the details of FUS phosphorylation *in vivo* are still under investigation, the involvement of two apical DDR kinases seems to suggest that protein aggregation and DDR activation might be mechanistically linked in causing neurodegeneration for a subset of ALS and FTD cases. Nevertheless, the consequence of phosphorylation on LLPS is protein- and residue specific. Indeed, Ser/Thr phosphorylation within FUS LCD reduces its aggregation *in vitro* and *in vivo* (Monahan et al., 2017), while it has the opposite effect on other proteins such as fragile-X linked protein FMRP (Tsang et al., 2019). Regarding TDP-43, phosphomimetic substitution with glutamic acid of serine 48 (Ser^48^Glu), a highly phosphorylated residue in the N-terminal domain (Rigbolt et al., 2011; Hornbeck et al., 2012; Hornbeck et al., 2015; Wang A. et al., 2018), also reduces LLPS, suggesting that phosphorylation of this residue interferes with the transient and weak intermolecular interactions necessary for phase transition, possibly promoting a more rigid and structured protein conformation (Wang A. et al., 2018). In line with this, different un-phosphorylatable mutations of Ser to Ala weakly induce phase transition. On the other hand, it is also known that phosphorylated TDP-43 represents one of the predominant components of protein aggregates in ALS and FTD (Hasegawa et al., 2008; Guedes et al., 2017), in which TDP-43 is found phosphorylated in its LCD, mainly on Ser 403/404 and 409/410 (Neumann et al., 2020). Nevertheless, whether this phosphorylation occurs before or after aggregation and its possible causative role in stabilizing protein aggregation need to be yet clarified. A possible explanation for these conflicting observations could be that phosphorylation can occur after TDP-43 aggregation, as an attempt to counteract detrimental interactions throughout electrostatic repulsions (Brady et al., 2011). Conversely, another theory that has been put forward proposes that phosphorylation prevents the clearance of the aggregates, thus causing their accumulation. In support of this, Zhang et al. have shown that phosphorylated fragments are more difficult to degrade than the non-phosphorylated ones (Zhang et al., 2010). Moreover, *in vitro* experiment casein kinases 1 (CK) increases TDP-43 phosphorylation and aggregation (Hasegawa et al., 2008).

Among all the factors that can influence both protein phosphorylation and aggregation, ATP concentration is strictly regulated within cells. On the one hand ATP plays an indirect role in controlling the assembly of condensates via protein phosphorylation; on the other hand, high ATP concentrations (>6 mM) alone can dissolve *in vitro-*generated liquid condensates of several RBPs, including FUS. The effect of ATP on LLPS directly stems from its hydrotropic nature, achieved due to the presence of the aromatic ring, capable of binding the hydrophobic patches in FUS (RGG and RRM domains), and the triphosphate chain that interacts with water molecules, thus leading to dissolution of protein aggregates (Patel et al., 2017; Rice and Rosen, 2017; Kang et al., 2019). It is worth noting that the cellular ATP concentration is usually in the millimolar range (up to 10 mM), while ADP and AMP are 50 and 10 µM respectively. The high ATP consumption of neurons may reduce its cellular concentration and might contribute to why these cell types are more prone than others to fibrillar degeneration of FUS condensates.

### Acetylation

While in other contexts of neurodegeneration protein acetylation has been widely associated with reduced protein aggregation (Saito et al., 2019), FUS and TDP-43 Lys acetylation leads to the formation of cytoplasmic protein aggregates (Table 1; Figures 2A,B).

Recently, three new acetylated Lys residues localized in different domains of FUS have been identified by mass spectrometric approaches (Arenas et al., 2020). In particular, acetylation at Lys315/Lys316 within the RRM domain strongly affects the ability of FUS of binding RNA, while acetylation of Lys510 in the NLS stimulates the formation of FUS-containing cytoplasmic aggregates (Arenas et al., 2020). Moreover, the application of a specific antibody directed against acetylated Lys510 (K510Ac) reveals a significant increase of acetylated FUS in ALS patients-derived dermic fibroblasts, suggesting the involvement of this PTM in FUS pathogenicity (Arenas et al., 2020). In this study, treatments with specific inhibitors proved that Lys510 acetylation is catalyzed by the CREB binding protein (CBP)/p300 (Figure 2B), while de-acetylation is carried out by both histone deacetylases (HDACs) and sirtuins (SIRTs) (Arenas et al., 2020). Although acetylation of FUS and TDP-43 seems to act preferably as a driving force for protein aggregates (Table 1), when occurring in specific positions it can work in the opposite direction.

Proteomics approaches have always identified the N-terminus of FUS as a preferential target of acetylation (Catherman et al., 2013; Rhoads et al., 2018b). Indeed, a recent study revealed a new FUS acetylation by the N-terminal acetyltransferases (NatA-NatF), confirmed by co-expression of recombinant FUS with the Nat A complex, which stimulates LCD LLPS without increasing the formation of aggregates (Bock et al., 2021). N-terminal acetylation is the addition of an acetyl group to the N-terminal amine group through an amide bond thus impeding protonation of the terminal amine reducing the propensity of the nearly uncharged FUS LCD domain to form aggregates.

Analogously, TDP-43 aggregation state is modulated by acetylation. It has been shown that upon sodium arsenite treatment, the CREB binding protein (CBP) acetylates TDP-43 on Lys145-Lys149. This modification impairs TDP-43 RNA binding ability and produces the accumulation of amyloid-like inclusions containing hyper-phosphorylated TDP-43 (Cohen et al., 2015). Interestingly, TDP-43 mutants bearing acetylation-mimic Lys to Gln substitution form cytosolic aggregates and exhibit other hallmarks of TDP-43 pathology (Wang et al., 2017).

As for arginine methylation in FUS, also TDP-43 tendency to acetylation appears different in ALS and FTD contexts, since the acetylated form of TDP-43 is detectable only in ALS spinal specimens and not in brain specimens from FTD-TDP-43 patients (Cohen et al., 2015), suggesting that this PTM might be a valuable specific biomarker to distinguish between these two pathologies.

### Ubiquitination

One of the cellular mechanisms involved in the clearance of misfolded protein is the ubiquitin-proteasomal system that functions as a “quality control” mechanism. In ALS, a remarkable fraction of ubiquitin (Ub) is sequestered into different types of inclusions (Leigh et al., 1991), thus reducing the pool of Ub available for physiological ubiquitination of different substrates during the execution of many cellular functions including transcription, DNA repair and signal transduction (Hershko and Ciechanover, 1998; Chen and Sun, 2009).

Some evidence suggests that FUS is recruited into ubiquitin-positive cytoplasmic inclusions. However, the ubiquitin-ligase responsible for FUS ubiquitination has not yet been described (Neumann et al., 2009; Deng et al., 2010; Seelaar et al., 2010; Farrawell et al., 2015). It has been proposed that, in a neuronal context, autophagy represents the preferred mechanism for the clearance of misfolded proteins (Nijholt et al., 2011). Recent studies have directly linked autophagy and protein aggregates and the new term “aggrephagy” has been coined to define the mechanism of aggregate clearance by autophagy (Øverbye et al., 2007). Nowadays the contribution of aggrephagy is widely investigated in the context of proteinopathies. Intriguingly, aggrephagy and autophagy compete for limited amounts of intermediate structures (e.g., phagophores) and this could cause reduced autophagy efficiency in resolving the aberrant aggregation of cytoskeleton proteins upon toxic induction (Larsen et al., 2002). On the other hand, it is plausible that the presence of ubiquitin in FUS-containing inclusions indicates an initial attempt to resolve protein aggregates via the proteasome degradation pathway (Farrawell et al., 2015).

Ubiquitination also has an important role in controlling the formation of TDP-43 condensates (Figure 2). Indeed, Lys48 and Lys63 ubiquitination by E3 ubiquitin ligase Parkin leads to cytosolic accumulation of TDP-43 and the formation of cytosolic condensates (Hebron et al., 2013). Interestingly, this re-localization reduces the Parkin mRNA level, which is in turn controlled by TDP-43. Overexpression experiments have proven the formation of a multi-protein complex comprising Parkin, ubiquitinated TDP-43 and HDAC6 that facilitates cytosolic accumulation of TDP-43. Although this cytosolic complex is likely to have a physiological function, a failure of the proteasome function in neurodegenerative diseases leads to the appearance of cytosolic TDP-43 condensates (Hebron et al., 2013). Notably, it has been suggested that the Parkin-mediated ubiquitination may contribute to TDP-43 aggregation (Hebron et al., 2013). It is worth noting that TDP-43 can be ubiquitinated by several other enzymes. An example is the UBE2E class of ubiquitin-conjugating enzymes that ubiquitylate TDP-43 at Lys263 (Hans et al., 2014). More recently, it has been reported that cytoplasmic inclusions resulted from the expression of two fALS mutants: TDP-43-Met^337^Val and FUS-Arg^495^x. These mutants co-localized with polymeric Ub^K63^, which is associated with the autophagy-related clearance mechanism. Intriguingly, the expression of FUS-Arg^495^x causes the reduction of monomeric ubiquitin levels that can disrupt ubiquitin homeostasis (Farrawell et al., 2020).

The level of ubiquitinated proteins within the cell is tightly regulated by deubiquitinating enzymes (DUBs), which counterbalance ubiquitin ligase activity and comprise a large family of enzymes with different specificities and catalytic activities. Due to their role in removing ubiquitin signaling, DUBs are implicated in a wide range of cellular processes and differentially accumulate in distinct functional compartments, based on their primary role (Clague et al., 2019). For instance, some cytosolic DUBs are coupled with the proteasome activity, and therefore may potentially be of major relevance in the modulation of protein aggregation state in the context of neurodegenerative disorders. Among these, Ubiquitin-specific protease 14 (USP14) is catalytically active only when bound to the 26S proteasome and contributes to the cleavage of ubiquitin from substrates before their degradation (Borodovsky et al., 2001; Hu et al., 2005). It has been shown that proteasome-associated USP14 deubiquitinates TDP-43 and that USP14 inhibition accelerates TDP-43 turnover. In particular, overexpression of WT USP14 in mouse embryonic fibroblasts leads to increased TDP-43 levels. This effect is abolished by both the expression of USP14 catalytically inactive form and USP14 small molecule inhibition (Lee et al., 2010), prompting the idea that ubiquitin chain trimming by USP14 might act as an antagonist of proteasome function.

It is worth noting, that DUBs have also been implicated in autophagy mechanisms. A genetic screen in *Drosophila* larval fat body identified Ubiquitin Iso-peptidase Y (UBPY), also called USP8, as a key player in the autophagy flux, whose RNAi-mediated silencing led to lysosomal defects and accumulation of malfunctioning autophagosomes (Jacomin et al., 2015). Notably, TDP-43 Lys^263^Glu mutant undergoes pathological hyper-ubiquitination and aggregation and if the ubiquitin-conjugating enzyme UBE2E3 actively ubiquitinates it, UBPY is able to counteract this PTM. The silencing of UBPY in fact enhances neurodegeneration in the retina of TDP-43 *Drosophila* ALS model system with accumulation of ubiquitinated and insoluble TDP-43. In this way, UBPY participates in the regulation of TDP-43 Lys^263^Glu solubility and exerts a neuroprotective function in *Drosophila melanogaster* (Hans et al., 2014). Moreover, recent results demonstrated that the Ubiquitin Specific protease 10 (USP10) positively regulates the stability of the autophagic protein LC3B, counterbalancing LC3B degradation and thus enhancing clearance of protein aggregates under stress conditions (Jia and Bonifacino, 2021).

Intriguingly, not only can DUBs modulate the protein aggregation state but they are also are recruited to membrane-less organelles or even have the ability to phase separate themselves. One example is the human USP42, which drives nuclear speckle phase separation dependent on its de-ubiquitinating activity, thus governing mRNA splicing events (Liu et al., 2021). In addition, it has been proven that other two human DUBs, namely USP5 and USP13, are recruited to stress granules shell and dictate their stabilization or disassembly through their activity of removing ubiquitin chains (Xie et al., 2018). Human USP5 also seems to target and regulate the expression of hnRNP-A1 (Vashistha et al., 2020), whereas the fly DUB Otu possess an LC domain that drives the formation of amyloid-like granules, which resemble FUS and hnRNP-A1 structures and are indispensable for Otu enzymatic activity (Ji et al., 2019).

Overall, while ubiquitinated RBPs seem to be generally associated with aggregates formation, the clearance of this form by means of DUBs could be useful in reversing or preventing these toxic events. Nevertheless, intricate networks, which are far from being fully understood, controls the cellular balance of protein ubiquitination and ubiquitin mediated signaling, which could contribute to fostering proteinopathies in neurodegenerative disorders.

### PARylation

Poly ADP-ribosylation (PARylation) is a reversible PTM in which ADP-ribose (ADPr) units are added to the Glu, Asp, Lys, Arg or Ser residues by poly (ADP-ribose) (PAR) polymerases (PARPs). This process is reversed by PAR glycohydrolase (PARG) (Slade et al., 2011). It has been shown that PARylation regulates the dynamics of the SGs and that PARP1 activation upon cell treatment with H_2_O_2_ markedly increases the level of PARylated hnRNP-A1 (Duan et al., 2019). Indeed, Lys298 (K298), immediately C-terminal of the M9 motif at the CTD of the protein (Figure 1) is a PARylation site. Interestingly, hnRNP-A1 also contains a PAR-binding motif, located between the two RRM domains (position 92–113, Figure 1). It mediates the interaction with PARylated proteins and its mutation abrogates the recruitment of hnRNP-A1 to stress granules. Decreased PARylation levels suppress the formation of SGs and the recruitment of hnRNP-A1 and TDP-43 to SGs, while higher PARylation levels delay the disassembly of SGs. Considering the close proximity between the PAR-binding motif and the RRMs in hnRNP-A1, their PARylation has an impact on the interaction with RNA. In addition, K298 PARylation regulates the nucleocytoplasmic transport of hnRNP-A1 and stress induced-K298 PARylation may serve as a nuclear export signal (Duan et al., 2019).

## Conclusive Remarks

Among different factors that can influence LLPS and its conversion to pathological aggregates, PTMs are now attracting a lot of interest for three main reasons: 1) they can accumulate and be modulated differently in aged and young individuals thus explaining why proteinopathies arise late in patients’ lives; 2) they could be used as biomarkers to define the pathology and detect its preclinical stage thus enabling early treatments; 3) they are ideal drug targets, and many compounds are already available which affect on protein PTMs and which could be repurposed for the treatments of specific proteinopathies. Thus, pharmacological approaches targeting PTMs could help dissolve pathological aggregates or prevent their formation, thus enhancing survival of neurons in affected individuals. In addition, the optimization of highly sensitive assays for the precise detection of PTM patterns associated with the disease might be a very useful for early diagnosis in personalized medicine. For example, mass spectrometry (MS) could be used to identify specific peptide patterns, associated with proteins PTMs that could delineate characteristic pathological profiles. Alternatively, antibodies could be generated against pathological PTM patterns and used in diagnosis. The main drawback of these approaches is the availability of useful specimens from patients, which could be very difficult to obtain as it would involve highly invasive techniques. Thus there is a need to identify diagnostic signatures in easily accessible tissues like blood or other body fluids. Another important point to consider is that the generation of antibodies that recognize PTMs in single protein positions may be challenging, since in some cases different proteins may present highly similar motifs and PTMs. Finally, costs and accessibility of MS methodologies might hinder their usage in routine diagnostic processes. In conclusion, we believe that deciphering the impact of PTMs on the formation of protein aggregates in different pathological contexts is the basis for setting up diagnostic and therapeutic tools in order to ameliorate the derived phenotypes.
